# Influence of Type I Interferons in Gammaherpesvirus-68 and Its Influence on EAE Enhancement

**DOI:** 10.3389/fimmu.2022.858583

**Published:** 2022-07-07

**Authors:** Ana Citlali Márquez, Carys Croft, Iryna Shanina, Marc Steven Horwitz

**Affiliations:** ^1^ Department of Microbiology and Immunology, University of British Columbia, Vancouver, BC, Canada; ^2^ BC Centre for Disease Control, University of British Columbia, Vancouver, BC, Canada; ^3^ Innate Immunity Unit, Institut Pasteur, Inserm U1223, Paris, France

**Keywords:** multiple sclerosis, type I interferons, Epstein-Barr virus, Experimental Autoimmune Encephalomyelitis (EAE), environmental factors

## Abstract

Epstein-Barr virus (EBV) has been identified as a putative trigger of multiple sclerosis (MS). Previously, we reported that mice latently infected with murine gammaherpesvirus 68 (γHV-68), the murine homolog to EBV, and induced for experimental autoimmune encephalomyelitis (EAE), developed an enhanced disease more reminiscent of MS. These prior results showed that expression of CD40 on CD11b^+^CD11c^+^ cells in latently infected mice was required to prime the strong Th1 response driving disease as well as decreasing Treg frequencies in the periphery and CNS. Subsequent work demonstrated that transfer of B cells from latently infected mice was sufficient to enhance disease. Herein, we show that B cells from infected mice do not need type I IFN signaling to drive a strong Th1 response, yet are important in driving infiltration of the CNS by CD8^+^ T cells. Given the importance of type I IFNs in MS, we used IFNARko mice in order to determine if type I IFN signaling was important in the enhancement of EAE in latently infected mice. We found that while type I IFNs are important for the control of γHV-68 infection and maintenance of latency, they do not have a direct effect in the development of enhanced EAE.

## Introduction

Multiple Sclerosis (MS) is a chronic inflammatory disease of the Central Nervous System (1). Considered an autoimmune disease, its causes have not been clearly elucidated. Nonetheless, it is widely accepted that the development of MS requires the combination of both genetic and environmental factors (2). One of the environmental factors with the strongest association to the development of MS is previous infection with Epstein Barr Virus (EBV), the agent causing infectious mononucleosis (3, 4). The exact mechanism describing how EBV is able to alter the immune response of the body, even years after primary infection, still needs to be determined. Previously, we described an animal model where C57Bl/6 mice infected with γHV68, a murine homologue of EBV (5), enhances the symptoms of Experimental Autoimmune Encephalomyelitis (EAE) (6). In this model mice show higher EAE scores as well as an enhanced Th1 response driven by CD11b^+^CD11c^+^ cells that leads to important infiltration of CD8^+^ T cells into the CNS, as well as higher production of IFNγ by CD4^+^ and CD8^+^ T cells, and downregulation of IL-17 and T regulatory cells (Tregs) (6, 7). In order to see EAE enhancement, it is indispensable that the virus is latent (7). Viral latency leads to the increase of phosphorylation of STAT1 and CD40 in CD11b^+^CD11c^+^ cells even before EAE induction (7). STAT1 phosphorylation plays a decisive role in the early stages of the activation of the IFNα receptor (IFNAR) (8). Although many other cytokines activate STAT1, it has been described that activation through IFNAR promotes antigen presentation and activation of T lymphocytes (8). Importantly, type 1 IFN production is necessary during γHV-68 infection in order to control reactivation and maintenance of latency (9). Interferon-beta has been widely used in the treatment of relapsing remitting-MS and has shown to be effective in reducing relapses in MS patients (10). Although interferon beta-1b treatment has limited success when used alone, it has a better effect when combined with other MS therapies (11).

Finally, we have shown that latently infected B cells are necessary in the development of enhanced symptoms of EAE and that they are most likely doing so by increased antigen presentation by B cells and by signaling to other antigen presenting cells (12). For these reasons, we propose that type I IFNs are important players in the communication between latently infected B cells and CD11b^+^CD11c^+^ cells that direct EAE enhancement. This suggests that EBV infection in humans can contribute to the upregulation of type I IFNs, helping on the development of MS. In this paper we explore the role of type I IFNs in the enhancement of EAE in mice latently infected with γHV-68.

Our results show that while type I IFNs are important for the control of γHV-68 infection and for the maintenance of latency of the virus, they do not affect EAE enhancement. Although type I IFNs might play an important role in the immune regulation of EAE and in the control of γHV-68, it is not indispensable for the enhancement of EAE symptoms in our model.

## Materials and Methods

### Mice and Ethics Statement

C57Bl/6 mice from the Jackson Laboratory and C57Bl/6 IFNARko were kindly donated by Dan Campell and were bred and maintained in the animal facility at the University of British Columbia. All animal work was performed in accordance with the regulation of the Canadian Council for Animal Care. The protocol was approved by the Animal Care Committee (ACC) of the University of British Columbia (Protocols A17- 0105, A17-018)

### Infections and EAE induction

Female and male mice between 8-10 weeks of age were infected intranasally (i.n.) with 100 pfu of γHV-68 WUMS strain (purchased from ATCC, propagated on BHK cells) or 15 μl of PBS as a control. EAE was induced at 5- 6 weeks post infection by injecting 100μl of emulsified Complete Freund’s Adjuvant (DIFCO) with 200μg of MOG _35-55_ (GenScript) and 400μg of desiccated Mycobacterium tuberculosis H37ra (DIFCO) subcutaneously. Mice also received two doses of 200ng of pertussis toxin (List Biologicals) *via* i.p. at the time of immunization and then again 48 hours later. EAE was assessed on a score from 0 to 5 as follows: 0, no clinical symptoms, 0.5 partially limp tail; 1, paralyzed tail; 2, loss of coordination; 2.5, one hind limb paralyzed; 3, both hind limbs paralyzed; 3.5, both hind limbs paralyzed accompanied by weakness in the forelimbs; 4, forelimbs paralyzed (humane endpoint); 5, moribund or dead.

### Viral Quantification

qPCR analysis of DNA samples was performed using 2x Quantitect Probe Mastermix (Qiagen, USA) on the Bio-Rad CFX96 Touch™ Real Time PCR Detection system. Primers, probes and gBlocks^®^ were obtained from Integrated DNA Technologies. Quantification of copies of mouse genome was done on 10ng of DNA by using primers and probe for a region of the mouse PTGER2 gene (Forward Primer: 5’ – TACCTTCAGCTGTACGCCAC – 3’; Reverse Primer: 5’ – GCCAGGAGAATGAGGTGGTC – 3’; Probe: 5’ –/56-FAM/CCTGCTGCT/ZEN/TATCGTGGCTG/3IABkFQ/– 3’) and absolutely quantified by use of a standard curve using concentrations from 5x10^7^ copies/ul to 5x10^1^ copies/ul. Quantification of copies of the γ-HV68 genome was done on 100ng of DNA by using primers and probe for a region of ORF50 (Forward Primer: 5’ –TGGACTTTGACAGCCCAGTA – 3’; Reverse Primer: 5’ – TCCCTTGAGGCAAATGATTC – 3’; Probe: 5’ –/56-FAM/TGACAGTGC/ZEN/CTATGGCCAAGTCTTG/3IABkFQ/– 3’) and absolutely quantified by use of a separate standard curve using concentrations from 2x10^4^ copies/ul to 2 copies/ul. Samples were run using a minimum of two technical replicates and all standard curves had an R^2^ greater than 0.95. The protocol was as follows: 95°C for 15 minutes, 95°C for 15s, 60°C for 1 min, repeated 50 times. Quantification of copy number was done using the CFX manager software. The ratio of virus genome copy number to mouse genome copy number was obtained using the following equation: $\frac{\#\text{copies of ORF}50}{\#\text{copies of }PTGER}\times \frac{2\;copies\;PTGER2/genome}{1copyORF50/genome}\;\left(simplified\;to\;\frac{\#\text{copies of ORF}50}{\#\text{copies of }PTGER}\times 2\right)$

### Immune Cells Isolation and Flow Cytometry

Mice were euthanized two weeks post EAE induction. They were perfused with 30cc of PBS, and brains, spinal cords, and spleens were isolated. A single cell suspension was generated from each organ. Immune cells from the CNS were isolated using a 30% Percoll gradient. For intracellular staining, CNS mononuclear cells, were stimulated for 4 hours in DMEM (Gibco) containing 10% FBS (Gibco), GolgiPlug (BD Biosciences), 10ng/ml PMA, and 500ng/ml ionomycin. Antibodies for the cell surface markers were added to the cells in PBS with 2% FBS for 30 min on ice. After washing, cells were resuspended in Fix/Perm buffer (eBiosciences) for 30-45 minutes on ice, washed twice, and incubated with Abs for intracellular antigens (cytokines and transcription factors) in Perm buffer (30 min, on ice). Fluorescently conjugated antibodies directed against CD4 (clone RM4-5), CD8 (clone 53-6.7), CD3 (clone eBio500A2), IFN-γ (clone XMG1.2), Foxp3 (clone FJK-16s), and IL-17 (eBio17B7) were all purchased from eBiosciences. Samples were acquired using a FACS LSR II (BD Biosciences) and analyzed with FlowJo software (Tree Star, Inc.).

### Adoptive Transfer

Spleens from IFNARko mice were isolated and a single cell suspension was obtained. Memory B cell enrichment was performed using a custom kit from STEMCELL that contained a combination of monoclonal antibodies, including IgD, as negative selection antibodies. After enrichment, cells were washed in blank DMEM and were adjusted to a concentration of 1-1.5 x10^6^ cells/200μl. Cells were injected IP into WT mice and, the following day, EAE was induced.

### Statistical Analysis

Results are reported as mean + standard error of the mean (SEM). Two-way ANOVA followed by Bonferroni’s correction for multiple comparisons was employed to compare EAE scores. Unpaired Student’s t-test was used for all other analyses (GraphPad Prism). A p Value of <0.05 was considered statistically significant ns > 0.05, * ≤ 0.05, ** ≤ 0.01, *** ≤ 0.001, **** ≤ 0.0001.

## Results

### γHV68 Establishes Latency at a Similar Rate in IFNARko and WT Mice

**Figure 1:**
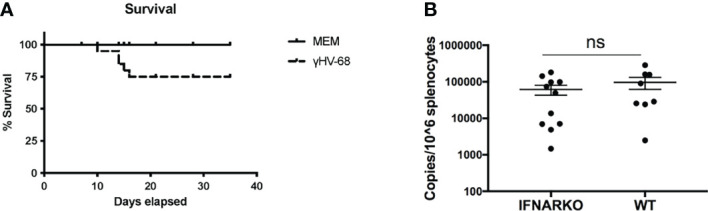
A low dose of γHV-68 is able to infect and establish latency in WT and IFNARko mice. **(A)** IFNARko mice were infected with 100 pfu of γHV-68. Survival rate in IFNARko mice was 75%. **(B)** 5 weeks p.i. IFNARko mice establish latency at similar rates than WT mice. Three independent experiments with 7-11 recipient mice/group. Data analyzed with Student’s t-test was not significant.

Both INFα and β signal through the receptor for IFNα/β (8). To study the effect of type I IFNs in the enhancement of EAE in mice latently infected with γHV-68, IFNARko mice, which lack the IFNα β receptor, are a useful tool. It has previously been described that IFNARko mice are able to control γHV-68 infection and successfully establish a latent infection in a similar proportion as WT mice (9). However, due to the lack of type I IFN response, the virus quickly overwhelms the mouse’s immune response and, at high doses, has a higher mortality rate during acute infection, not allowing the establishment of latency (9). Prior work by Barton et al. demonstrated that a 100pfu dose of γHV-68 given intranasally reduced mortality in IFNARko mice. We found that this regimen resulted in a survival rate of 75% (**Figure 1A**) and allowed the immune system to control the acute infection while still letting the virus establish latent infection. Quantification of latency with this low dose shows a slightly lower number of copies in IFNARko mice compared to WT, however this difference was not statistically significant (**Figure 1B** **)**. These results are similar to what has been previously described by Barton (9).

### γHV-68 Latently Infected IFNARko Mice Develop Enhanced T Cell Infiltration Into the CNS

The presence of Type I IFNs has been associated with a protective effect in MS patients. One of the first lines of treatment for MS is IFN-beta. However, as many as 30-50% of patients do not respond to the treatment (13). The mechanism for why this happens has not been described yet. Here, we explored whether the absence of type I IFN receptor had any effect on the ability of γHV-68 to drive the infiltration of immune cells into the CNS when IFNARko mice were latently infected with γHV-68.

**Figure 2:**
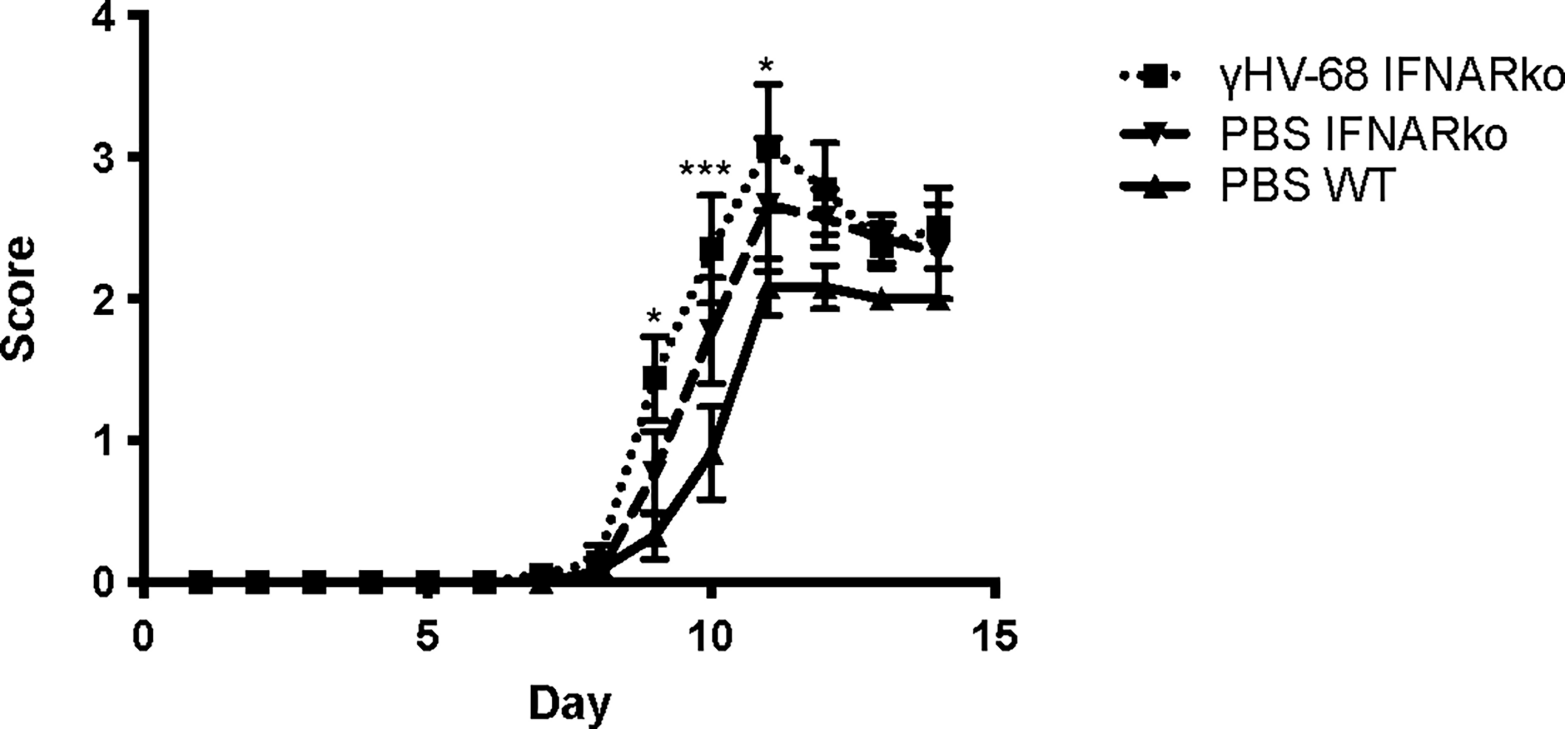
γHV-68 latently infected IFNARko mice develop enhanced EAE symptoms. IFNARko mice were infected with γHV-68. Five weeks p.i. EAE was induced. Graph shows EAE scores up to day 14 post induction in IFNARko mice and in γHV-68 IFNARko infected mice as well as WT uninfected mice (MEM). Statistical comparison shown is between PBS WT mice and IFNAko Two independent experiments 4-11 mice/group. Data analyzed with two-way ANOVA test with Bonferroni’s correction for multiple comparisons: ***p<0.001, *p<0.05.

**Figure 3:**
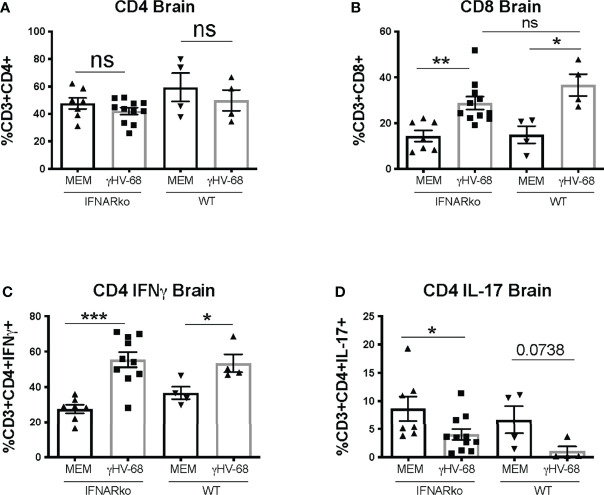
γHV-68 latently infected IFNARko mice develop enhanced Tcell infiltration into the CNS. IFNARko mice were infected with γHV-68. Five weeks p.i. EAE was induced. Mice were harvested at day 14 post EAE induction they were perfused, and brain, spinal cords and spleens were harvested and stained. Percentage of **(A)** CD3^+^CD4^+^, **(B)** CD3^+^CD8^+^ infiltrating cells in the brain and **(C)** CD3^+^CD4^+^IFNg^+^ and **(D)** CD3^+^CD4^+^IL-17^+^ in the brain. Two separate experiments with 4-11 mice/group. Data analyzed with Student’s t-test: ***p<0.001, **p<0.01, *p<0.05.

Previously, we reported that latent γHV-68 leads to a strong infiltration of CD4^+^ and CD8^+^ T cells in the CNS during EAE, and this T cell response is heavily skewed towards a Th1 phenotype (6). We infected IFNARko mice with γHV-68 and waited 5 weeks to allow for the establishment of latency. After this time, EAE was induced. As expected from previous reports (14), IFNARko mice demonstrated a slightly clinically enhanced EAE compared to WT uninfected mice although the difference is not significant. However, IFNARko mice showed a higher clinical score when they are infected with γHV-68 and there is a significant difference in score compared to WT uninfected mice (**Figure 2** **and** **Supplementary Figure 1**). Uninfected IFNARko mice show limited infiltration of T cells to the brain, and most of these cells are CD4^+^. On the contrary, IFNARko mice latently infected with γHV-68 show high levels of T cells infiltrating the brain (**Figures 3A, B** **)** and spinal cord (not shown), in particular CD8^+^ T lymphocytes. Remarkably, the amount of T cell infiltration in the CNS of γHV-68 IFNARko mice equals the one from γHV-68 WT mice. This is accompanied by a strong production of IFNγ and a downregulation of IL-17 **(** **Figures 3C, D** **)**. This T cell infiltration and cytokine profile is comparable to what we had previously observed in WT mice latently infected with γHV-68, which suggests that T cell infiltration and/or cellular enhanced infiltration into the CNS does not depend on the presence of Type I IFNs.

### Treg Proportion Is Affected by Type 1 IFNs During γHV-68 Latent Infection

**Figure 4:**
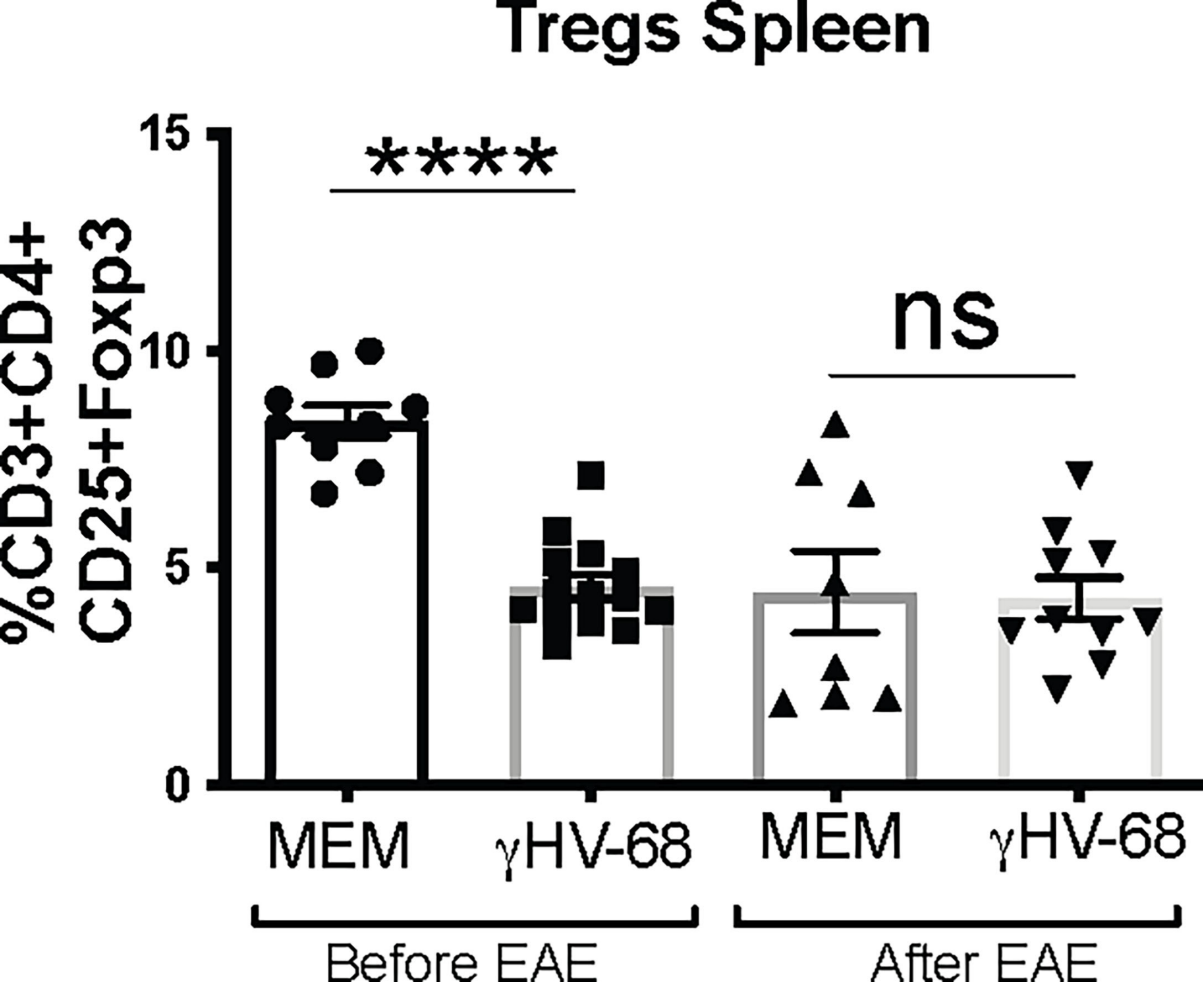
Tregs from γHV-68 latently infected IFNARko mice are downregulated before and after EAE induction. IFNARko mice were infected with γHV-68. 5 weeks p.i. mice were induced or not with EAE, they were harvested at day 14 post induction, spleens were harvested and stained. Percentage of CD3^+^CD4^+^CD25^+^Foxp3^+^ in the spleen. n= 8-10/group 2 separate experiments. Data analyzed with Student’s t-test: ****p<0. 0001.

Both our lab and others (Gasper-Smith 2006, Casiraghi 2015) have described that latent infection of γHV-68 leads to the downregulation of Tregs in the CNS and in the periphery. This downregulation is sustained after EAE induction. It has also been shown that depletion of Tregs during EAE exacerbates symptoms by facilitating an upregulation of cytokine production by T cells (15). We observed a marked downregulation in the number of Tregs in the periphery of IFNARko during γHV-68 latency (**Figure 4** **and** **Supplementary Figure 2** **)**; interestingly, although downregulation of Tregs is sustained after EAE induction in latently infected mice, Tregs are also downregulated in uninfected IFNARko mice **(** **Figure 4** **)**.

### B Cells Infected With γHV-68 Require Type I IFNs to Direct CD8 T Cell Infiltration in the CNS

**Figure 5:**
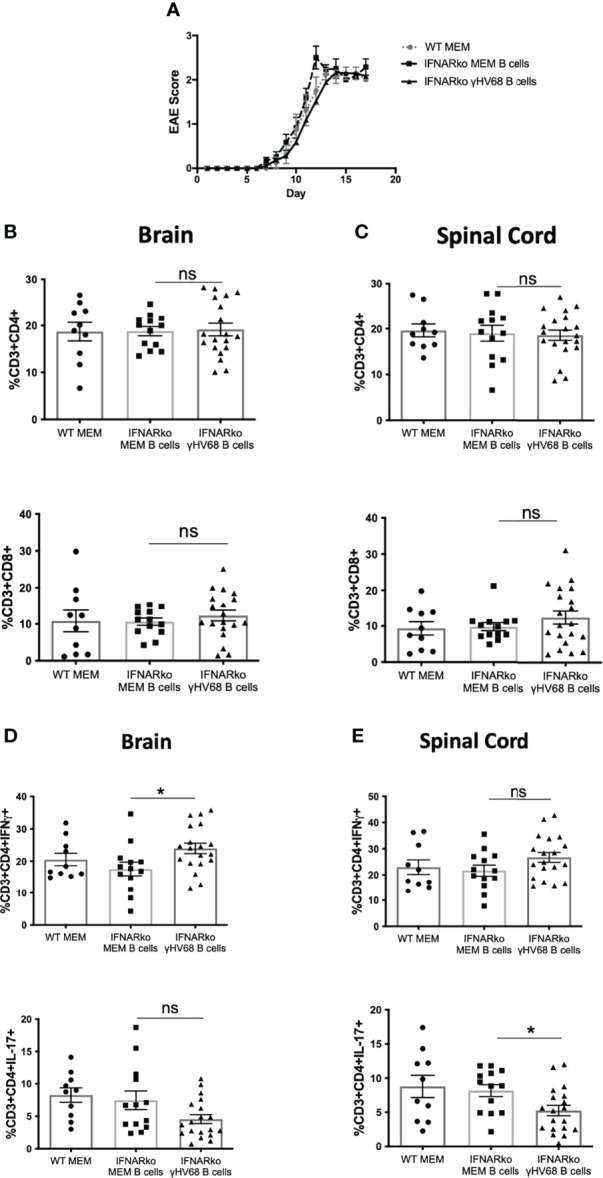
Memory B cells from IFNARko/WT mice infected with γHV-68 or not, were transferred to uninfected WT mice. 24 hours after the transfer, EAE was induced. At days 16-18 post EAE induction, mice were perfused; brains and spinal cords were harvested and processed to isolate immune infiltrates. **(A)** EAE scores up to day 18 post induction. **(B)** CD4^+^ and CD8^+^ infiltrating cells in the brain and **(C)** spinal cord. **(D)** Percentage of CD4^+^IFNg^+^ and CD4^+^IL-17^+^ in the brain and **(E)** spinal cord. Four independent experiments with 11-20 mice/group. Data analyzed with Student’s t-test: *p<0.05.

Finally, we tested whether type I IFN signaling was necessary to transfer disease into WT mice. Previously we showed that enriched CD19^+^ IgD^-^ B cells from γHV-68 mice, which are the main reservoir of γHV-68 during latency, are able to transfer EAE enhancement into naïve mice (12). When we transfer the CD19^+^IgD^-^ B cells from infected IFNARko mice into WT mice, we find that B cells from IFNARko mice infected with γHV-68 do not drive a strong infiltration of CD8^+^ T cells into the CNS **(** **Figures 5B, C** **)**. Interestingly, B cells from γHV68 mice have a slight effect in the production of IFNγ and downregulation of IL-17 **(** **Figure 5D, E** **)**. This suggests that while type I IFNs are directly responsible of skewing a strong Th1 response they might have a role in the infiltration of CD8 T cells into the CNS.

## Discussion

Type I IFNs have been identified as an important therapeutic target because of their protective role in MS and EAE development (16–19). IFN-α has also shown effectiveness in reducing MS relapses, although it is less effective than β-IFN (20). This suggests that while type I IFNs can modify MS, other factors are involved in the modification of the disease. Infection with pathogenic agents that otherwise would be innocuous to the organism might override the protective effect of type I IFNs and could lead to the development of MS relapses.

This paper explores the potential role of type I IFNs in mice infected with a homolog of EBV - γHV-68. Using the model we previously developed (6), we showed that despite the importance of type I IFN signaling in the control and latency of the virus, there is still a strong Th1 response in IFNARko mice that leads to an EAE enhancement similar to the one observed in γHV-68 WT mice (**Figures 1**, **2**). This suggests that type I IFNs are dispensable for the enhancement of EAE symptoms. It is very possible that even if type I IFNs are helping to lead EAE enhancement, other factors are involved that require the combination of different cytokines and chemokines produced by the B cell in order to activate the CD11b^+^CD11c^+^ cells. This activation directs a strong Th1 response that ultimately will develop EAE enhancement and immune cell infiltration into the CNS independent of Type I IFNs.

Type I IFN upregulation during viral infection has been associated with an inhibition of Treg activation and proliferation (21). IFNARko mice have a downregulation in Tregs, which could help to explain why these mice show enhanced EAE symptoms. Contrary to what was expected, Tregs remain at low levels during latent infection with γHV-68.

Finally, adoptive transfers suggest, that even though B cells from infected mice do not need type I IFN signaling to drive a strong Th1 response, they do seem to be important in directing CD8^+^ T cell infiltration in the CNS. It has been suggested that type I IFNs can provide the “third signal” that is necessary for the activation and clonal expansion of CD8 T cells (22). It is possible that IFNs delivered directly by B cells are important in the activation of cells that will infiltrate the CNS, although more experiments need to be done to confirm this.

Overall, this paper demonstrates that despite the importance of type I IFN in regulating the immune response during acute viral infection and the development of EAE, it does not have an effect in the enhancement of EAE symptoms caused by latent infection with γHV-68, highlighting a type I IFN independent pathway. These results can help to understand why the effectiveness of interferon beta-1b can be so unpredictable in MS patients and suggests that EBV status might be important in the predicting the effectiveness of treatment. Additional experiments need to be done to explore the role of γHV-68/EBV latency in EAE and MS, in particular, the role of memory cells, the main reservoir of virus during latency.

## Data Availability Statement

The original contributions presented in the study are included in the article/**Supplementary Material**. Further inquiries can be directed to the corresponding author.

## Ethics Statement

The animal study was reviewed and approved by Animal Care Committee (ACC) of the University of British Columbia (Protocols A17- 0105, A17-018).

## Author Contributions

ACM and MH conceived and designed the experiments. ACM, CC, and IS conducted the experiments. ACM and MH analyzed the results and wrote the manuscript. All authors contributed to the article and approved the submitted version.

## Funding

This work was supported by a grant from the MS Society of Canada to MH and ACM received a PhD fellowship from the MS Society of Canada and Consejo Nacional de Ciencia y Tecnología (CONACyT).

## Conflict of Interest

The authors declare that the research was conducted in the absence of any commercial or financial relationships that could be construed as a potential conflict of interest.

## Publisher’s Note

All claims expressed in this article are solely those of the authors and do not necessarily represent those of their affiliated organizations, or those of the publisher, the editors and the reviewers. Any product that may be evaluated in this article, or claim that may be made by its manufacturer, is not guaranteed or endorsed by the publisher.

## Supplementary Material

Supplementary Figure 1 Representative plots of T cell infiltration into the CNS in IFNARko mice.

Supplementary Figure 2 Gating strategy for immune cell infiltrates to the central nervous system.

Supplementary Figure 3 Gating strategy for Treg cells in the spleen
