# Supplementary figures and images for: Influence of Type I Interferons in Gammaherpesvirus-68 and Its Influence on EAE Enhancement
Source: Front Immunol. 2022 Jul 7;13:858583. doi: 10.3389/fimmu.2022.858583 (PMC9301468; doi:10.3389/fimmu.2022.858583)

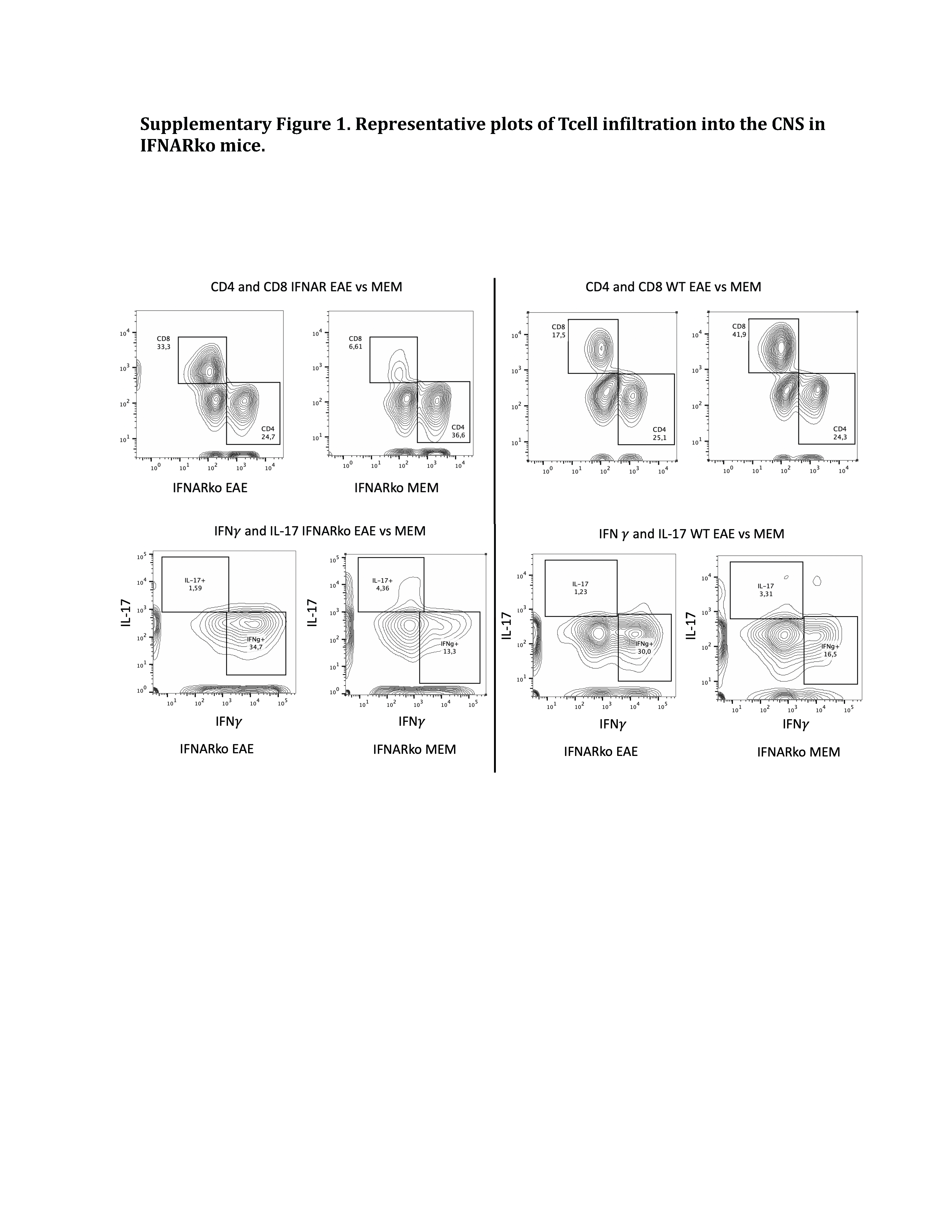

Supplement: Supplementary Figure 1 — Representative plots of T cell infiltration into the CNS in IFNARko mice. [file Image_1.tiff]

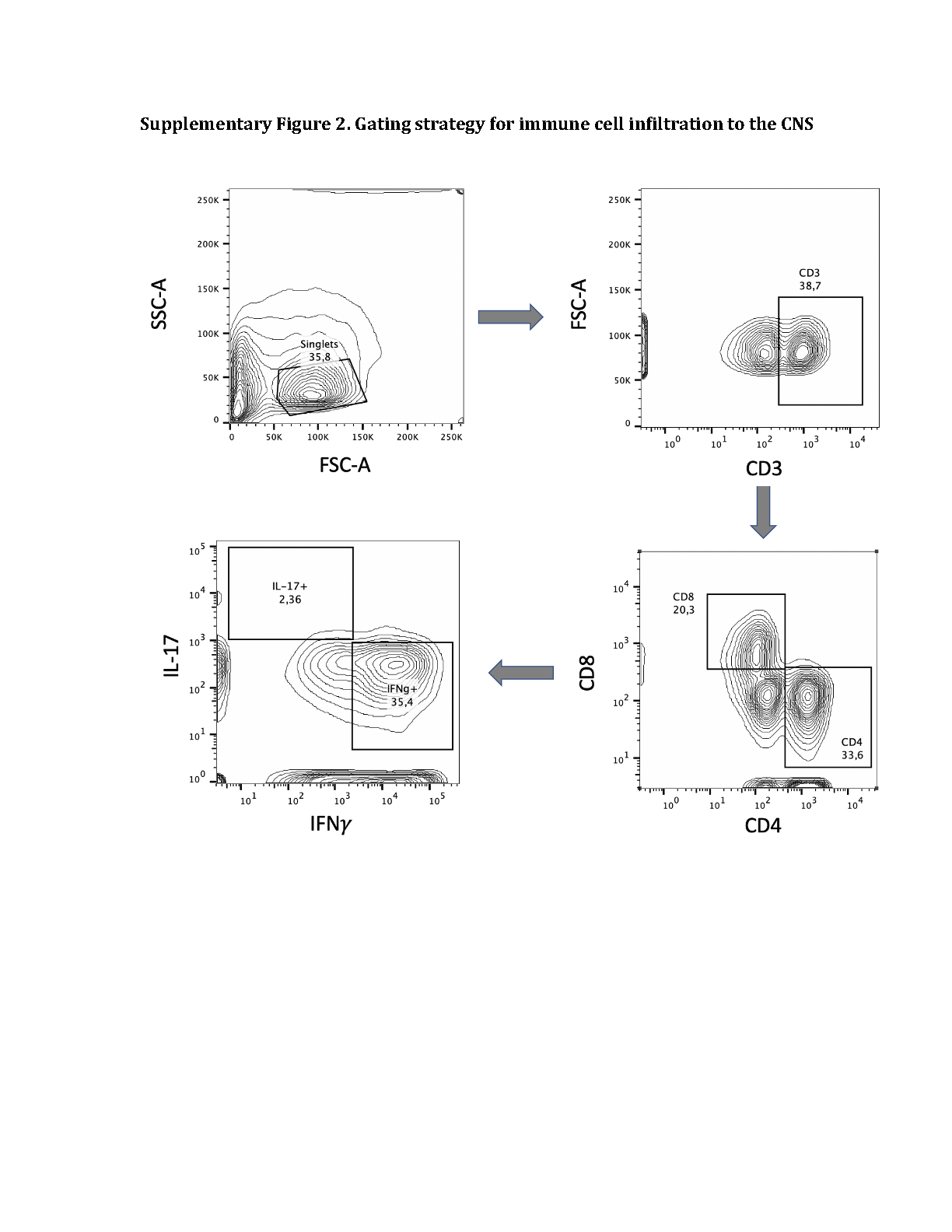

Supplement: Supplementary Figure 2 — Gating strategy for immune cell infiltrates to the central nervous system. [file Image_2.tiff]

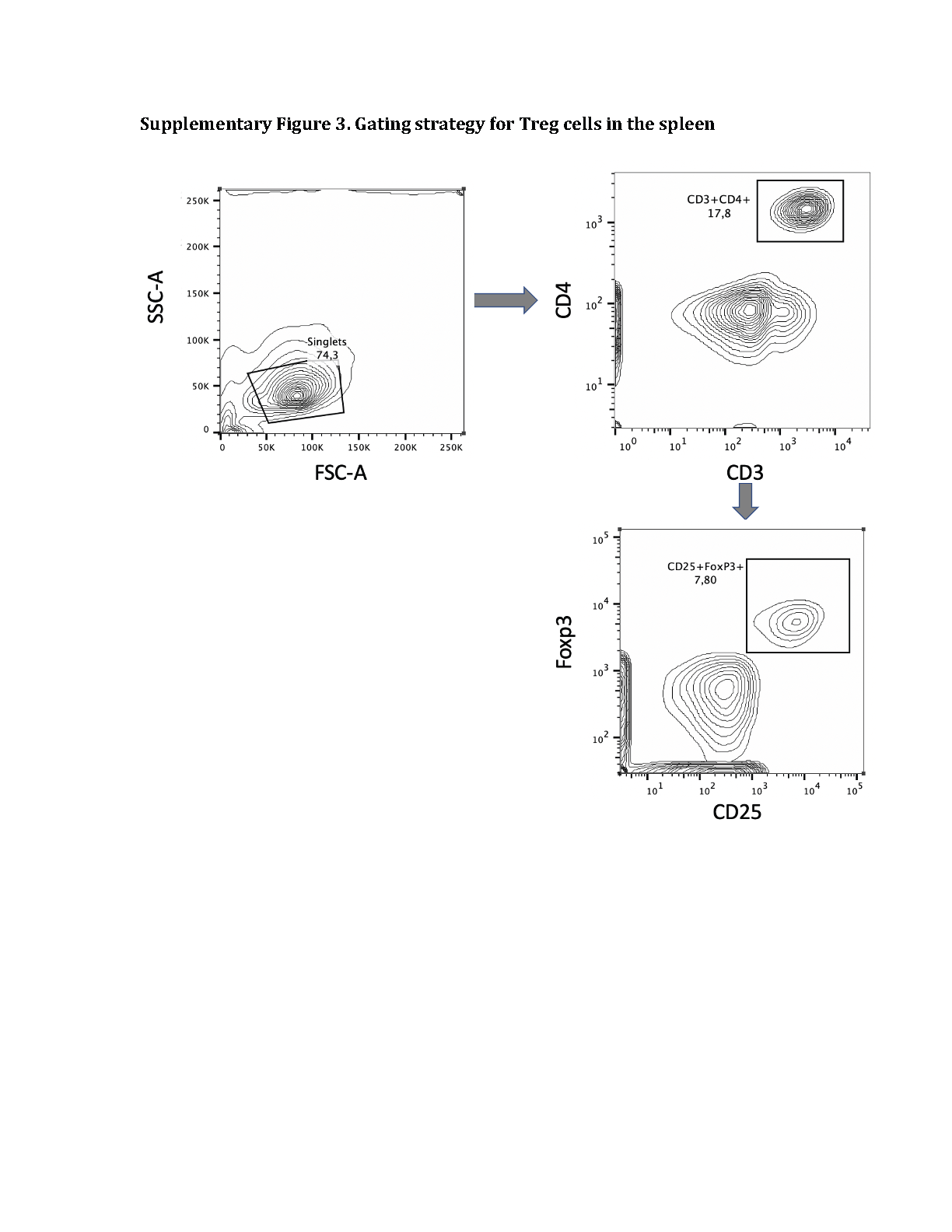

Supplement: Supplementary Figure 3 — Gating strategy for Treg cells in the spleen [file Image_3.tiff]
